# BDNF/TrkB signalling, in cooperation with muscarinic signalling, retrogradely regulates PKA pathway to phosphorylate SNAP-25 and Synapsin-1 at the neuromuscular junction

**DOI:** 10.1186/s12964-024-01735-2

**Published:** 2024-07-23

**Authors:** Aleksandra Polishchuk, Víctor Cilleros-Mañé, Marta Balanyà-Segura, Laia Just-Borràs, Anton Forniés-Mariné, Carolina Silvera-Simón, Marta Tomàs, Meryem Jami El Hirchi, Erica Hurtado, Josep Tomàs, Maria A. Lanuza

**Affiliations:** 1 Universitat Rovira i Virgili. Unitat d’Histologia i Neurobiologia (UHNeurob), Facultat de Medicina i Ciències de la Salut. c/ Sant Llorenç 21, Reus, 43201 Spain; 2 Unitat d’Histologia i Neurobiologia (UHNeurob), Institut d’Investigació Sanitària Pere Virgili (IISPV), Reus, Spain

**Keywords:** Motor endplate, Acetylcholine release, Muscarinic receptors, BDNF, TrkB, Serine kinases, PKA

## Abstract

**Background:**

Protein kinase A (PKA) enhances neurotransmission at the neuromuscular junction (NMJ), which is retrogradely regulated by nerve-induced muscle contraction to promote Acetylcholine (ACh) release through the phosphorylation of molecules involved in synaptic vesicle exocytosis (SNAP-25 and Synapsin-1). However, the molecular mechanism of the retrograde regulation of PKA subunits and its targets by BDNF/TrkB pathway and muscarinic signalling has not been demonstrated until now. At the NMJ, retrograde control is mainly associated with BDNF/TrkB signalling as muscle contraction enhances BDNF levels and controls specific kinases involved in the neurotransmission. Neurotransmission at the NMJ is also highly modulated by muscarinic receptors M_1_ and M_2_ (mAChRs), which are related to PKA and TrkB signallings. Here, we investigated the hypothesis that TrkB, in cooperation with mAChRs, regulates the activity-dependent dynamics of PKA subunits to phosphorylate SNAP-25 and Synapsin-1.

**Methods:**

To explore this, we stimulated the rat phrenic nerve at 1Hz (30 minutes), with or without subsequent contraction (abolished by µ-conotoxin GIIIB). Pharmacological treatments were conducted with the anti-TrkB antibody clone 47/TrkB for TrkB inhibition and exogenous h-BDNF; muscarinic inhibition with Pirenzepine-dihydrochloride and Methoctramine-tetrahydrochloride for M_1_ and M_2_ mAChRs, respectively. Diaphragm protein levels and phosphorylation’ changes were detected by Western blotting. Location of the target proteins was demonstrated using immunohistochemistry.

**Results:**

While TrkB does not directly impact the levels of PKA catalytic subunits Cα and Cβ, it regulates PKA regulatory subunits RIα and RIIβ, facilitating the phosphorylation of critical exocytotic targets such as SNAP-25 and Synapsin-1. Furthermore, the muscarinic receptors pathway maintains a delicate balance in this regulatory process. These findings explain the dynamic interplay of PKA subunits influenced by BDNF/TrkB signalling, M_1_ and M_2_ mAChRs pathways, that are differently regulated by pre- and postsynaptic activity, demonstrating the specific roles of the BDNF/TrkB and muscarinic receptors pathway in retrograde regulation.

**Conclusion:**

This complex molecular interplay has the relevance of interrelating two fundamental pathways in PKA-synaptic modulation: one retrograde (neurotrophic) and the other autocrine (muscarinic). This deepens the fundamental understanding of neuromuscular physiology of neurotransmission that gives plasticity to synapses and holds the potential for identifying therapeutic strategies in conditions characterized by impaired neuromuscular communication.

**Supplementary Information:**

The online version contains supplementary material available at 10.1186/s12964-024-01735-2.

## Background

Protein kinase A (PKA), a serine-threonine protein kinase activated by Cyclic adenosine monophosphate (cAMP), regulates neurotransmission at various synapses, including the neuromuscular junction (NMJ), where PKA activity enhances Acetylcholine (ACh) release [1, 2]. Recently, it has been demonstrated that, at the NMJ, nerve stimulation regulates PKA activity in neurotransmission, and the resulting nerve-induced muscle contraction retrogradely modulates it, contributing to plasticity [3]. This highlights the molecular mechanism facilitating activity-dependent bidirectional communication between nerve terminals and muscle cells, ensuring the precision of ACh release. There is information about the connection of PKA with other molecular pathways as BDNF/TrkB or muscarinic signallings [1, 4–7], but the molecular interplay between these pathways and PKA subunits and targets during presynaptic activity or nerve-induced muscle contraction is remained unclear. To date, the cellular communication, bidirectionally occurring at the NMJ, has been linked primarily to the neurotrophic Brain-derived neurotrophic factor (BDNF)/Tropomyosin-related kinase B receptor (TrkB) signalling pathway and the presynaptic Protein kinase C (PKC) pathway [8–10]. BDNF by binding to the TrkB, actively participates in the retrograde neuroprotective regulation mediated by muscles, which is essential for maintaining motor neuron function. Muscle contraction elevates BDNF levels both in vitro and in vivo [10, 11], and the subsequent BDNF/TrkB signalling plays a retrograde regulatory role on presynaptic isoforms of PKC and the machinery governing neurotransmission [8–10]. The TrkB receptor exhibits three isoforms through alternative splicing: TrkB.T1 (truncated isoform 1), TrkB.T2 (truncated isoform 2), and TrkB.FL (full-length isoform). TrkB.FL supports neural cell survival, whereas TrkB.T1, lacking the tyrosine kinase domain, shows a dominant negative effect on TrkB.FL signalling [12, 13]. TrkB.T2, also lacking the kinase domain, is mainly absent at the NMJ [14–16]. At the NMJ, it has been shown that activity-dependent alterations in the BDNF/TrkB signalling occur through adjustments in the TrkB isoform ratio. Muscle contraction downregulates TrkB.T1, elevating the TrkB.FL/TrkB.T1 ratio and increasing TrkB.FL activity [10]. This shift in the TrkB.FL and TrkB.T1 balance directly influence the neurotrophic regulation of ACh secretion and could explain the activity-dependent effects observed in the PKA pathway, as PKA also appears to be a promising candidate for the regulation of neurotransmission via BDNF/TrkB signalling [4, 5]. In the short term, PKA consistently enhances ACh release at the NMJ [1, 2] and modulates the conductivity of neural ion channels [17]. In the long term, PKA exerts influence on Acetylcholine receptor (AChR) stability at the NMJ [18], modulates the mRNA translation rate of specific synaptic proteins [19, 20], and participates in the regeneration of the NMJ [21]. Moreover, PKA collaborates with PKC to regulate ACh release at the NMJ [2, 22], and PKA has been associated with BDNF/TrkB retrograde signalling during NMJ development [6] and in amyotrophic lateral sclerosis (ALS) disease [23].

The modulation of neuromuscular transmission by BDNF/TrkB signalling includes interactions with presynaptic muscarinic acetylcholine receptors (mAChRs) [24] but their molecular relationship in the NMJ is still unclear. At the NMJ, only M_1_ and M_2_ subtypes of mAChRs are functional, with M_1_ enhancing and M_2_ decreasing ACh release, respectively [1, 25–27]. We know that in the basal conditions mAChRs regulate presynaptic regulatory and catalytic PKA subunits’ dynamics to phosphorylate PKA-targets [7], like SNAP-25 and Synapsin-1 that are key presynaptic targets of PKA and are involved in regulating ACh release. PKA phosphorylation of SNAP-25 at Threonine 138 (T138) [28, 29] induces a reduction in SNARE (SNAP Receptors) complex stability, facilitating the dynamic processes of synaptic vesicle recycling and refilling, which are essential for effective neurotransmission [30]. Synapsin-1 plays a critical role in connecting synaptic vesicles to the actin cytoskeleton [31]. When phosphorylated by PKA at Serine 9 (S9) [32, 33], Synapsin-1 undergoes dissociation from the vesicle membrane and this enables vesicles to diffuse to the nerve terminal periphery, thereby amplifying their recycling rate [31, 34]. Here, we hypothesize that the interplay between BDNF/TrkB signalling and mAChRs cooperatively regulates the dynamic behaviour of PKA subunits, promoting their catalytic activity at the NMJ, and this activation leads to the phosphorylation of key targets involved in synaptic vesicle exocytosis (SNAP-25 and Synapsin-1). Our findings demonstrate that BDNF/TrkB signalling differentially regulates the PKA pathway depending on the synaptic activity conditions and that mAChRs are involved on it. These discoveries contribute novel insights into the activity-dependent bidirectional communication between nerve terminals and muscle fibres, highlighting the role of PKA-phosphorylation targets in enhancing ACh release at the NMJ and interrelating two fundamental pathways in synaptic modulation: one retrograde (neurotrophic) and the other autocrine (muscarinic). This deepens the fundamental understanding of neuromuscular physiology of neurotransmission that gives plasticity to synapses and holds the potential for identifying therapeutic strategies in conditions characterized by impaired neuromuscular communication.

## Methods

### Animal care

Adult Sprague Dawley rats of both genders (30–40 days old; Criffa, Barcelona, Spain; RRID: RGD_5508397) were maintained in compliance with the humane treatment guidelines outlined in the European Community Council Directive for laboratory animals. For tissue collection, animals were humanely euthanized with a lethal dose of 4% tribromoethanol (Sigma-Aldrich). Each distinct experimental condition included a minimum of three animals (*n* ≥ 3) as biological replicates. All procedures involving animals received approval from the Ethics Committee of Animal Experimentation at Universitat Rovira i Virgili, under reference number 10760.

### Antibodies

For the Western blotting experiments conducted in this study, primary and secondary antibodies were obtained from the specified providers, and their concentrations are detailed in Table 1. Prior to the implementation of the Western blotting technique, we verified the specificity of primary antibodies, confirming specific bands at their respective molecular weights in accordance with expectations [3].

**Table 1:** Antibodies

Target	Epitope	Source	Company (#cat)	Dilution
Cα	Hu Cα C-terminus	Ms mAb	Santa Cruz (sc-28,315)	1/1000
Cβ	Hu Cβ C-terminus	Rb pAb	Santa Cruz (sc-904)	1/1000
RIα	Hu RIα residues 1-381	Ms mAb	Santa Cruz (sc-136,231)	1/1000
RIβ	Hu RIβ C-terminus	Rb pAb	Santa Cruz (sc-907)	1/1000
RIIα	Ms RIIα C-terminus	Rb pAb	Santa Cruz (sc-909)	1/1000
RIIβ	Hu RIIβ residues 21–110	Ms mAb	Santa Cruz (sc-376,778)	1/800
SNAP-25	Hu SNAP-25 residues around Gln116	Rb mAb	CST (5309)	1/1000 1/500
pSNAP-25 (T138)	Hu SNAP-25 residues around T138	Rb pAb	Biorbyt (orb163730)	1/1000
Synapsin-1	Hu Synapsin-1a, b	Rb pAb	Chemicon (AB1543)	1/1000 1/500
pSynapsin-1 (S9)	Hu Synapsin-1 residues around S9	Rb pAb	CST (2311 S)	1/1000
AKAP150	Rat AKAP150 residues 428–449	Rb pAb	Millipore (07-210)	1/1000
mAChR M_1_	Hu M_1_ mAChR residues within 227–353 (3rd intracellular loop)	Rb pAb	Alomone Labs (AMR-001)	1/1000
mAChR M_2_	Pig mAChR M_2_	Ms mAb	Santa Cruz (sc-80,971)	1/1000
S100 (β-Subunit)	Epitope located on the β-chain (i.e., in S-100a and S-100b)	Ms mAb	Sigma-Aldrich (S2532)	1/50
Secondary antibodies	Anti-Rb conjugated HRP	Dk pAb	Jackson ImmunoResearch (711-035-152)	1/10,000
	Anti-Ms conjugated HRP	Rb pAb	Sigma-Aldrich (A9044)	1/10,000
	Anti-Ms conjugated TRITC	Dk pAb	Jackson ImmunoResearch (715-025-151)	1/1000
	Anti-Rb conjugated Alexa fluor 488	Dk pAb	Invitrogen Antibodies (A-21,206)	1/1000
	α-Bungarotoxin conjugated Alexa Fluor 647		Thermo Scientific (B35450)	1/800

Note: Antibodies used in this study and procedure specifications

Abbreviations: Dk, donkey; Hu, human; mAb, monoclonal antibody; Ms, mouse; pAb, polyclonal antibody; Rb, rabbit

### Reagents

In order to block muscle contraction, we utilized µ-conotoxin GIIIB (#C-270, Alomone Labs Ltd, Jerusalem, Israel). This particular toxin effectively inhibits sarcolemmal Voltage-dependent sodium channels (VSDCs) while leaving synaptic ACh release and ACh signalling unaffected [2, 35]. The toxin was supplied in a lyophilized powder form with a purity exceeding 99%. We prepared a stock solution of µ-conotoxin GIIIB at a concentration of 150 µM, and the working concentration used in Ringer’s solution (mM: NaCl 137, KCl 5, CaCl2 2, MgSO4 1, NaH_2_PO_4_ 1, NaHCO_3_ 12, glucose 12.1, and DMSO 0.1%) was 1.5 µM. This solution was oxygenated with a mixture of O2 and CO2 in a ratio of 95:5.

The anti-TrkB antibody clone 47/TrkB (#610102, BD Transduction Laboratories) was employed for conducting TrkB inhibition assays, with a working solution prepared at 10 mg/ml concentration. Additionally, exogenous incubations involving BDNF were carried out using h-BDNF (#B-250, Alomone Labs Ltd, Jerusalem, Israel) at a concentration of 10 mM.

To estimate the effect of muscarinic inhibition, we have applied Pirenzepine dihydrochloride (PIR) (#1071, Tocris), M_1_ mAChR selective agonist: 10 mM stock and used at 10 µM; Methoctramine tetrahydrochloride (MET) (#M105, Sigma), M_2_ mAChR selective agonist: 1 mM stock and used at 1 µM.

In both control and drug-containing conditions all chemicals were diluted in Ringer’s solution contained 0.1% dimethyl sulfoxide (DMSO) as the vehicle.

### Tissue dissection and treatment

The diaphragm muscle, a commonly used model for investigating the development and functionality of the NMJ [36–38], was carefully dissected to maintain phrenic nerve connectivity. Isolated nerve-muscle preparations were then preserved in Ringer’s solution and maintained at a constant temperature of 26 °C.

One hemidiaphragm was dedicated to the experimental treatment, while its counterpart remained untreated for comparative analysis in this ex vivo experimental setup. Muscle contraction was achieved through phrenic nerve stimulation at a frequency of 1 Hz. This frequency was chosen to maintain various tonic functions while avoiding synaptic vesicle depletion. Stimulation was administered for a duration of 30 min, facilitated by the A-M Systems 2100 isolated pulse generator (A-M System), according with previous research methodologies [8–10].

**Fig. 1:**
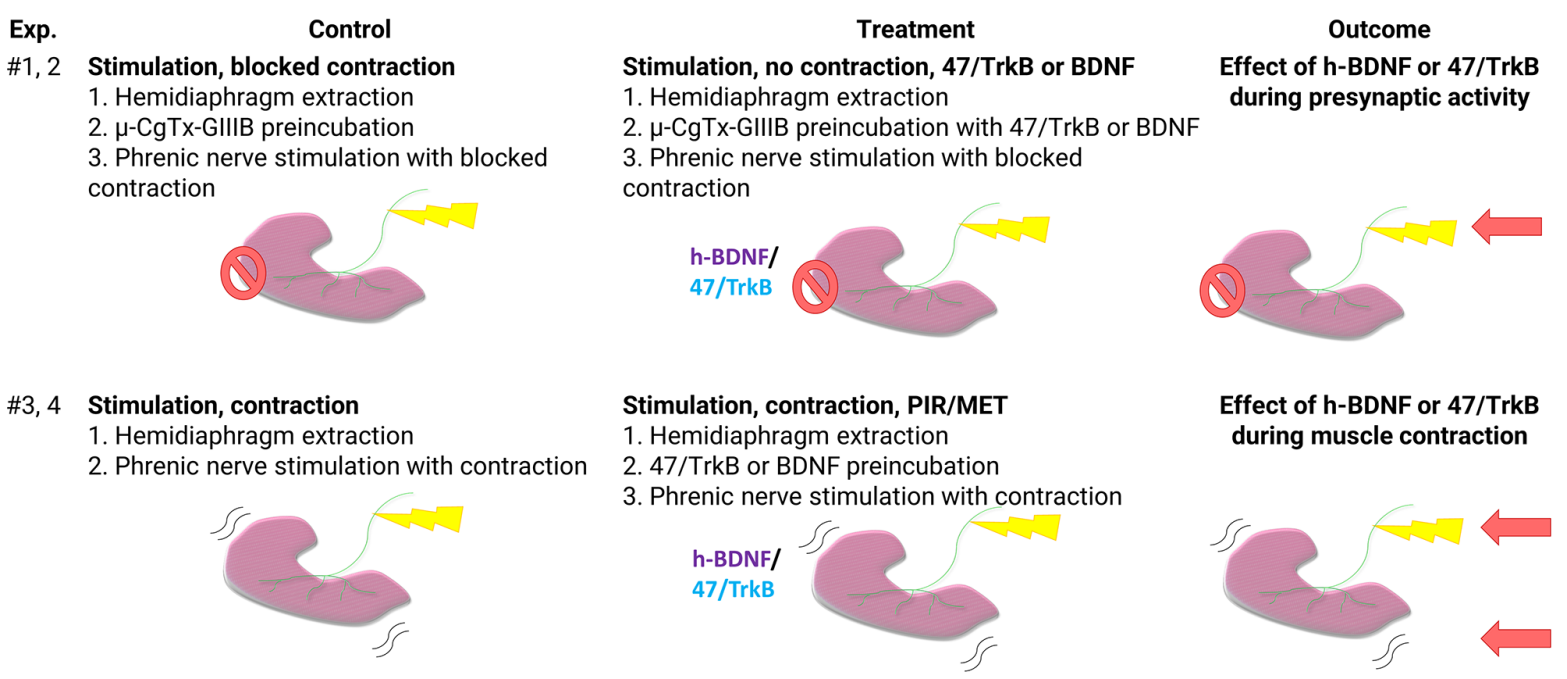
Experimental design for investigating the impact of exogenous h-BDNF addition or TrkB inactivation during presynaptic activity and nerve-induced muscle contraction. µ-CgTx-GIIIB, µ-conotoxin GIIIB; h-BDNF, exogenous recombinant human Brain-Derived Neurotrophic Factor; 47/trkB, anti-TrkB antibody clone 47/TrkB

Our stimulation protocol was designed to maintain nerve stimulation and the associated neurotransmission mechanisms, effectively preventing non-nerve-induced (direct) muscle contraction mechanisms [39–41]. Visual inspection was applied to verify muscle contraction. In our study, we conducted four main experiments to distinguish the effects of exogenous h-BDNF and blockade of TrkB receptor during synaptic activity or nerve-induced muscle contraction, as illustrated in Fig. 1.

1. To estimate the effect of exogenous h-BDNF under synaptic activity we compared presynaptically stimulated muscles whose contraction was blocked by µ-CgTx-GIIIB with and without BDNF: ES (electrical stimulation) vs ES + h-BDNF.
2. To demonstrate the influence of TrkB inhibition during synaptic activity, we compared muscles stimulated presynaptically, with contraction blocked by µ-CgTx-GIIIB, both with and without 47/TrkB: ES vs ES + 47/TrkB.
3. To show the impact of the exogenous h-BDNF under muscle contraction, we compared stimulating and contracting muscles with and without BDNF: (ES + C) (electrical stimulation with muscle contraction) vs (ES + C) + BDNF.
4. To demonstrate the effects of TrkB inhibition during muscle contraction, we compared muscles that were stimulated and contracted with and without 47/TrkB: (ES + C) vs (ES + C) + 47/TrkB.

In experiments where stimulation without muscle contraction was required, we applied µ-CgTx-GIIIB (see Reagents section). However, prior to immersing these muscles in µ-CgTx-GIIIB, we conducted a visual inspection to confirm the accurate contraction of the muscle [9].

**Fig. 2:**
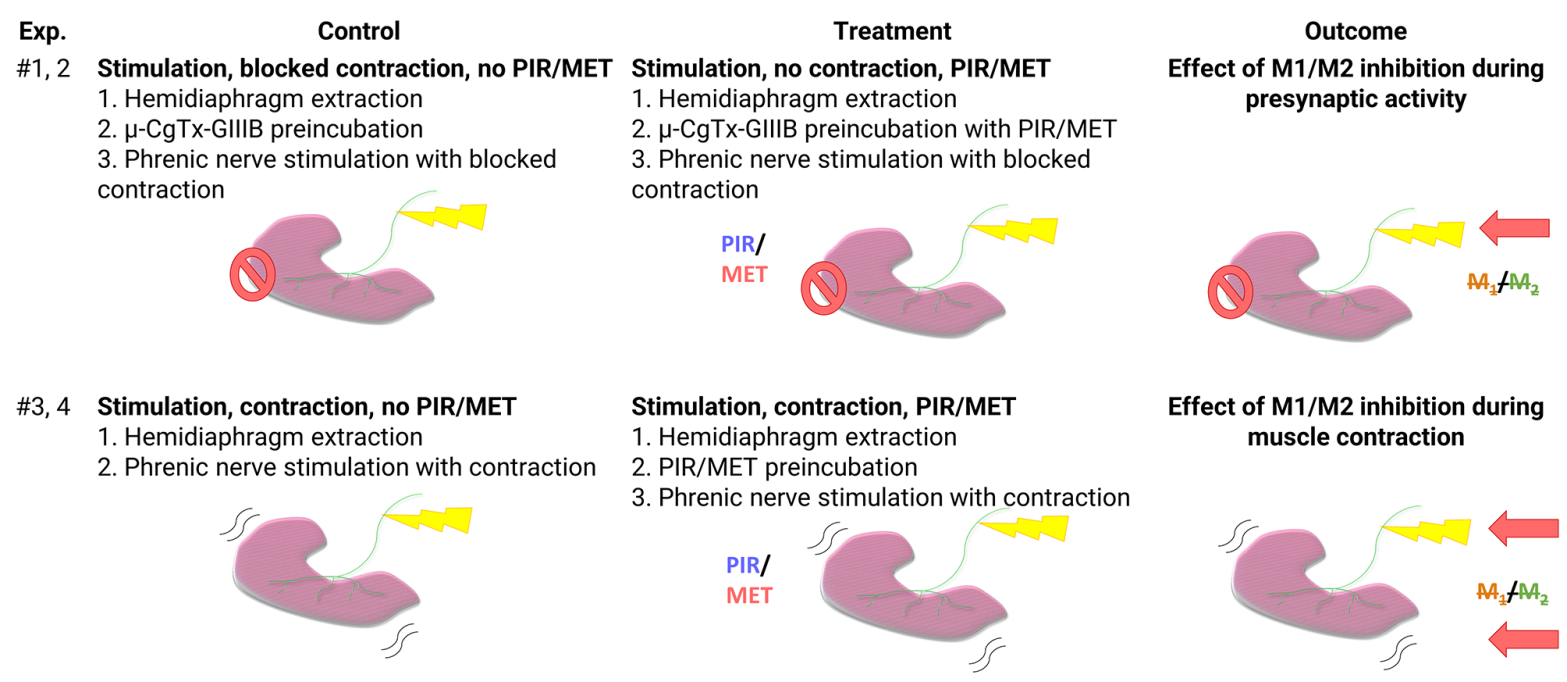
Design of experimental treatment for the study of effects of M_1_ or M_2_ mAChRs inhibition during presynaptic activity and nerve-induced muscle contraction. µ-CgTx-GIIIB, µ-conotoxin GIIIB; PIR, Pirenzepine dihydrochloride (M_1_ mAChR selective agonist); MET, Methoctramine tetrahydrochloride (M_2_ mAChR selective agonist)

To investigate possible effects of the muscarinic pathway on the targets of PKA SNAP-25 and Synapsin-1 we have performed the following experiments (Fig. 2):

1, 2) To assess the influence of M_1_/M_2_ mAChR on PKA targets during synaptic activity, we compared presynaptically stimulated muscles with inhibited contraction by µ-CgTx-GIIIB, with and without PIR or MET, selective antagonists for M_1_ and M_2_ mAChR, respectively: ES vs ES + PIR/MET.

3, 4) To show the impact of inhibiting M_1_/M_2_ mAChR on PKA targets during muscle contraction, we compared muscles undergoing stimulation and contraction with and without PIR or MET, respectively: (ES + C) vs (ES + C) + PIR/MET.

### Sample processing by Western blotting and fractionation

Upon completion of the treatment, whole cell lysate samples were immediately frozen. For more detailed information regarding the homogenization and Western blotting techniques applied, please refer to Cilleros-Mañé et al., 2021 [22].

The analysis of band densitometry was conducted using ImageJ 1.52a software (National Institutes of Health, USA) [42]. The integrated optical density of the bands was normalized against both background values and the total protein transferred onto PVDF membranes, which was evaluated through total protein analysis using Sypro Ruby protein blot stain from Bio-Rad [43].

Relative variations between the experimental and control samples were assessed based on the same membrane image. The data presented in this study are based on densitometry measurements obtained from 3 to 10 individual replicates, with comparisons made against control samples. It’s important to note that the data quantification was conducted blindly.

### Immunohistochemistry

We conducted immunohistochemical (IHC) localization of Synapsin-1 and SNAP-25 within the *levator auris longus* (LAL) muscle, following the methodology outlined in Cilleros-Mañé et al., 2021 [22]. For this analysis, we used LAL muscle samples harvested from the same animals that were previously used in the Western blot experiments (*n* = 3).

As part of our control measures, some muscle samples underwent immunohistochemical procedures in which primary antibodies were omitted. These control muscle specimens displayed no evidence of positive staining. When applying double-staining protocols, the omission of either one of the two primary antibodies led to the complete elimination of the corresponding staining, with no observed cross-reaction between the other primary antibody. Our experimental setup included a minimum of three muscle samples utilized as negative controls to ensure the validity of our findings.

We used the Zeiss LSM880 AiryScan confocal microscope to examine immunolabeled NMJs in whole-mount muscles [22]. Images were taken with a Zeiss PlanApo Å~63 1.42 NA oil objective. Subsequently, 3D colocalization analyses were conducted on confocal stacks using FIJI (ImageJ 1.54f) software (National Institutes of Health, USA) [44]. For the quantitative assessment of colocalization, we utilized the Pearson correlation coefficient (r), which provides an overall measure of the association between two probes in an image. Representative images were assembled for display with Adobe Photoshop software 8.0.1 (Adobe Systems, San Jose, CA).

### Statistical analysis

For every experiment previously detailed, we ensured a minimum of three animals (*n* ≥ 3) served as biological replicates. As a result, all the experiments were carried out with a minimum of three biological replicates, and each of these was subject to assessment in three technical replicates. The determination of the sample size, with the goal of optimizing the use of animals, was based on criteria established in previous studies [45, 46].

The results are displayed in the form of ratios or percentages comparing treatment outcomes to control, and they are presented as the mean ± SEM (Standard Error of the Mean). We assessed sample normality using the Shapiro-Wilk test and determined significant differences using either the paired Student’s t-test or the non-parametric Wilcoxon test, both performed using GraphPad Prism version 8.0.2 for Windows (GraphPad Software, Boston, Massachusetts USA).

In the figures, each dot found within the bars corresponds to the mean result obtained from an individual animal. We denoted statistical significance using the following thresholds: *p < 0.05, **p < 0.01, and ***p < 0.001.

## Results

### Presynaptic activity-induced BDNF/TrkB pathway regulates PKA catalytic and regulatory subunits, Synapsin-1, SNAP-25 and AKAP-150 protein levels

The PKA holoenzyme is composed of two Regulatory (R) and two Catalytic (C) subunits, which form an inactive tetramer. Murine models express four isoforms of the R subunit (RIα, RIβ, RIIα, RIIβ) and two isoforms of the C subunit (Cα, Cβ) [47, 48] and dynamics between R and C subunits determine the PKA activity.

**Fig. 3:**
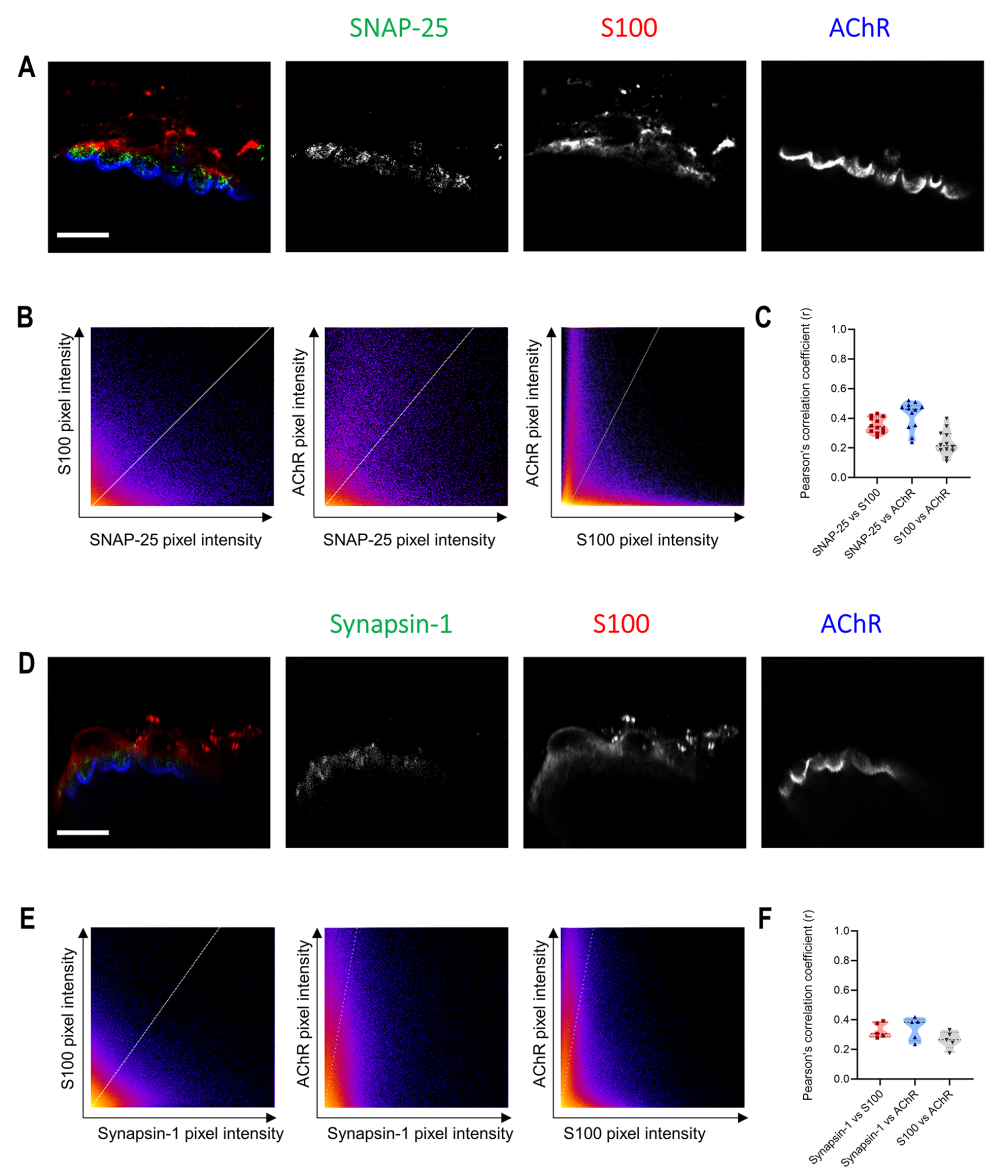
SNAP-25 and Synapsin-1 localization at the adult NMJ. **A** Confocal image of a NMJ with triple labelling: SNAP-25 (green), S100 (red), and AChR (blue). Scale bar: 10 μm. **B** Example of quantitative colocalization represented as heatmap of the intensity of between anti-SNAP-25 labelling, Schwann Cell anti-S100 labelling and postsynaptic AChR labelling. **C** Average Pearson’s correlation coefficient of the colocalization between SNAP-25 versus S100, SNAP-25 versus AChRs and S100 versus AChR. **D** Confocal image of a NMJ with triple labelling: Synapsin-1 (green), S100 (red), and AChR (blue). Scale bar, 10 μm. **E** Example of quantitative colocalization represented as heatmap of the intensity of between anti-Synapsin-1 labelling, Schwann Cell anti-S100 labelling and postsynaptic AChR labelling. **F** Average Pearson’s correlation coefficient of the colocalization between Synapsin-1 versus S100, SNAP-25 versus AChRs and S100 versus AChR.

As PKA is present in the three cellular components of the NMJ, to determine its presynaptic activity, we studied PKA substrates exclusively expressed in motor neurons: Synapsin-1 and SNAP-25 [3]. Here, we demonstrate the absence of colocalization with the Schwann cell marker S100. On the first hand, SNAP-25 immunolabel was only found in the middle of the sandwich formed by glia and ACh receptors (Fig. 3A). Immunohistochemistry analysis revealed scarce colocalization between SNAP-25 and the Schwann cell marker S100 (*r* = 0.35 ± 0.05) and between SNAP-25 and AChR (*r* = 0.42 ± 0.10) (Fig. 3B-C). This indicates a precise localization of SNAP-25 within the neural tissue. Similarly, the Synapsin-1 labelling appeared in the areas between S100 marker (*r* = 0.33 ± 0.05) and postsynaptic AChRs with almost no overlapping (*r* = 0.34 ± 0.08) (Fig. 3D). That was also confirmed with image analysis (Fig. 3E-F).

To identify how both neurotrophic control and presynaptic activity interact on the PKA at the NMJ, we applied presynaptic electrical stimulation (ES) in conjunction with TrkB receptor inhibition (ES + 47/TrkB) or exogenous h-BDNF incubation (ES + h-BDNF).

**Fig. 4:**
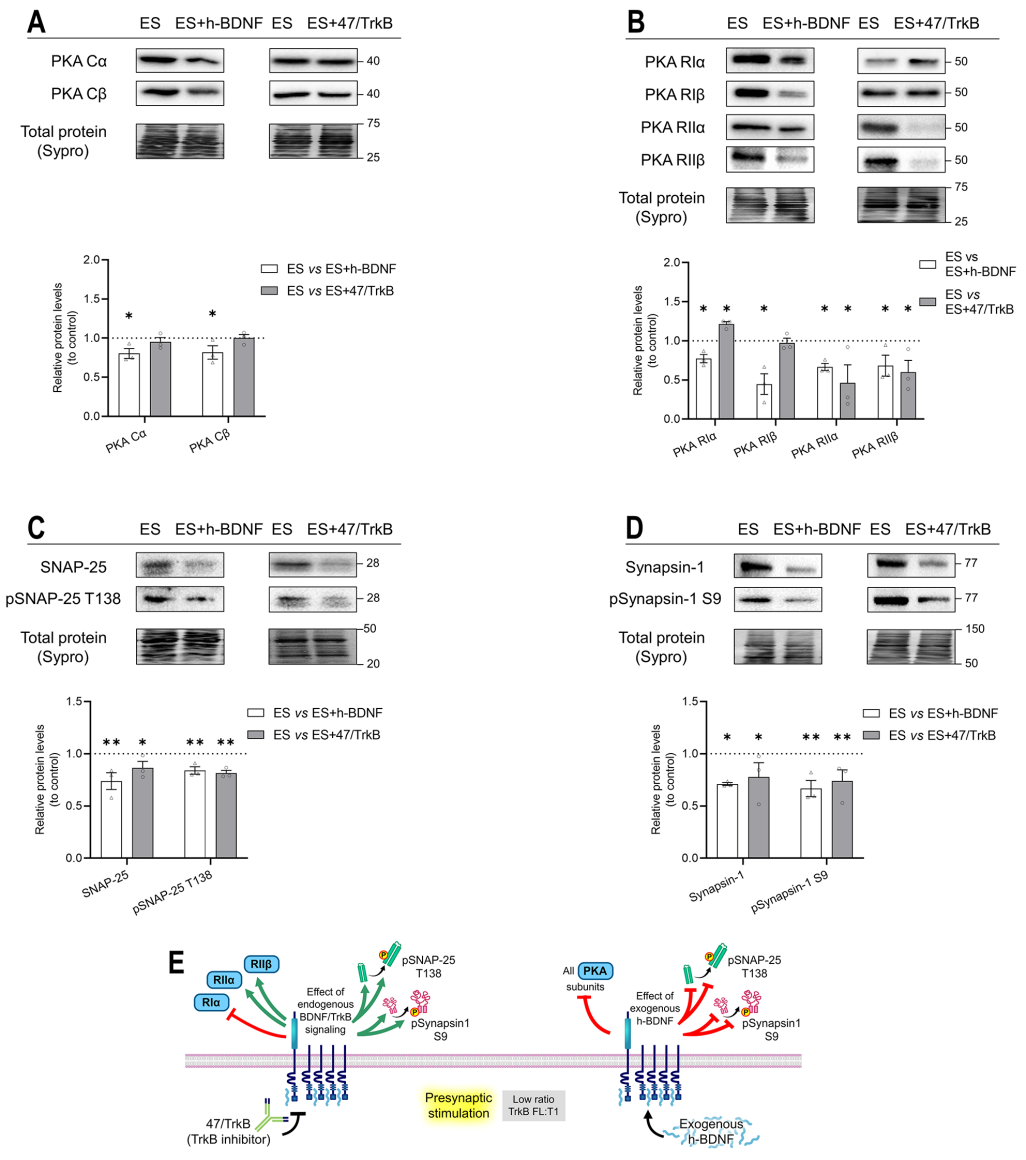
Modulation by exogenous h-BDNF or 47/TrkB of PKA catalytic and regulatory subunits and its targets SNAP-25 and Synapsin-1 protein levels during presynaptic activity. **A-D** Western blot analysis of protein levels after treatment with presynaptic stimulation with h-BDNF —ES versus ES + h-BDNF— and with 47/TrkB —ES versus ES + 47/TrkB. **A** Catalytic PKA Cα and Cβ subunits. **B** Protein kinase A regulatory subunit Iα/Iβ/IIα/IIβ. **C** SNAP-25 and its phosphorylated form pSNAP-25 T138. **D** Synapsin-1 and its phosphorylated form pSynapsin-1 S9. Data are expressed relative to experimental control – ES (dotted line) (mean ± SEM). *p < 0.05, **p < 0.01, and ***p < 0.001 versus the corresponding control. **E** Graphical representation of the results, effect of endogenous TrkB activity and exogenous h-BDNF during presynaptic stimulation on PKA subunits and targets. ES, electrical stimulation; Cα/β, Protein kinase A Catalytic subunit α/β; RIα/RIβ/RIIα/RIIβ, Protein kinase A Regulatory subunits Iα/Iβ/IIα/Iiβ; h-BDNF, exogenous recombinant human Brain-Derived Neurotrophic Factor; 47/trkB, anti-TrkB antibody clone 47/TrkB

The addition of 47/TrkB in the presynaptic stimulus condition (ES vs ES + 47/TrkB) did not affect PKA Cα nor PKA Cβ levels (Fig. 4A). On the other hand, exogenous h-BDNF (ES vs ES + h-BDNF) decreased Cα and Cβ levels.

The PKA regulatory subunits showed different trends (Fig. 4B). TrkB blockade decreased RIIα and RIIβ, increased RIα, and did not affect RIβ. These results demonstrate different relationships between the regulatory PKA subunits and the BDNF/TrkB receptor pathway. Exogenous h-BDNF decreased all the regulatory subunits (Fig. 4B).

Regarding PKA substrates, SNAP-25 (Fig. 4C) and Synapsin-1 (Fig. 4D) responded equally to the neurotrophic modulations. Both TrkB blockade and exogenous h-BDNF decreased the total and PKA-phosphorylated forms of SNAP-25 and Synapsin-1 during the presynaptic stimulus condition.

To facilitate understanding of the results Fig. 4E shows a graphical abstract for interpretation.

**Fig. 5:**
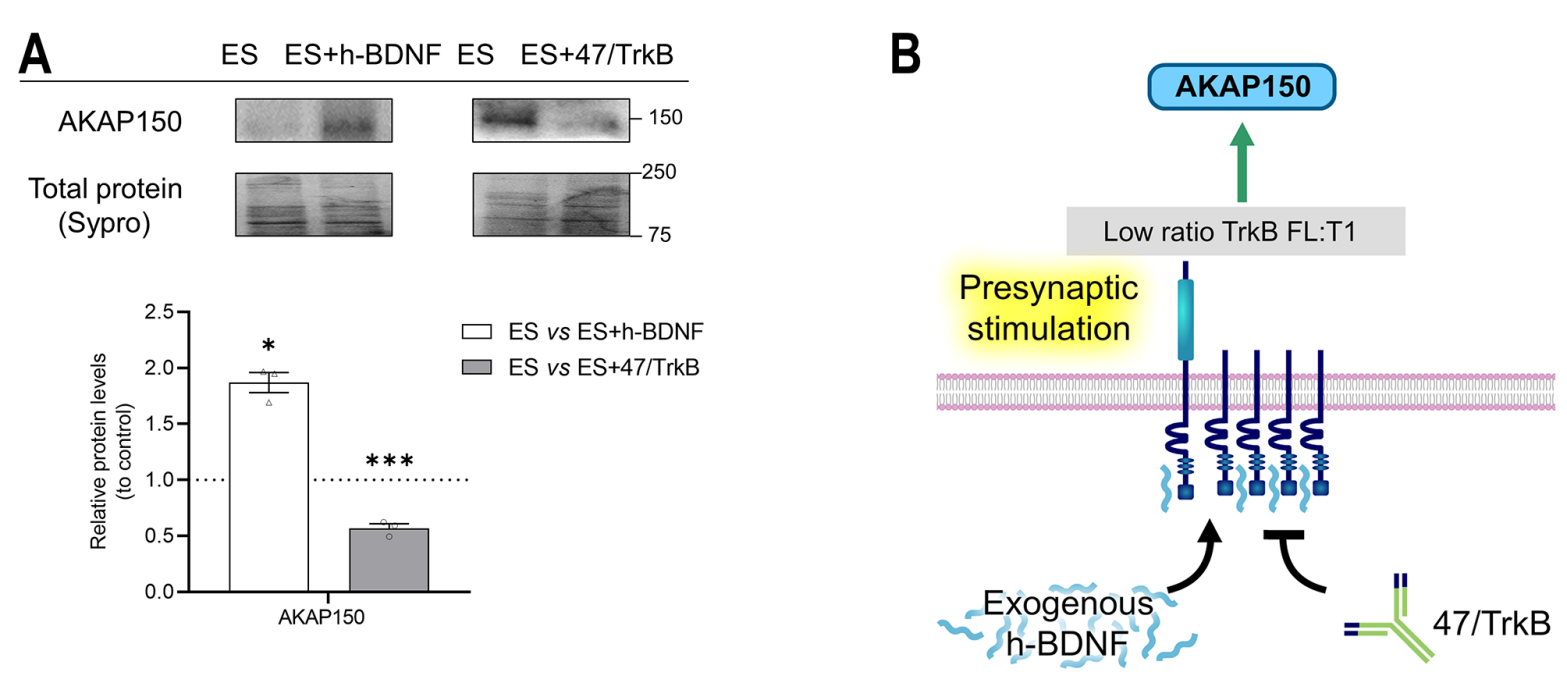
Modulation by exogenous h-BDNF or 47/TrkB of AKAP150 protein levels during presynaptic activity. **A** Western blot analysis and data quantification of AKAP150 protein levels in the diaphragm muscle after presynaptic activity with h-BDNF or 47/TrkB incubation. Data are expressed relative to experimental control – ES (dotted line) (mean ± SEM). *p < 0.05, **p < 0.01, and ***p < 0.001 versus the corresponding control. **B** Graphical representation of the results, effect of endogenous TrkB activity and exogenous h-BDNF during presynaptic stimulation on AKAP150. AKAP150, A-kinase anchor protein 150; ES, electrical stimulation; h-BDNF, exogenous recombinant human Brain-Derived Neurotrophic Factor; 47/trkB, anti-TrkB antibody clone 47/TrkB

We found that AKAP150 (A-Kinase Anchoring Protein 150) under presynaptic stimulus conditions is decreased by TrkB blockade and increased by exogenous h-BDNF (Fig. 5A). Figure 5B shows a graphical summary of the findings.

In summary, catalytic subunits (Cα and Cβ) and regulatory subunit RIβ remain unaltered after TrkB inhibition. In contrast, RIIα and RIIβ, AKAP150, and the targets Synapsin-1 and SNAP-25 decreased indicating that they need TrkB to maintain their presynaptic activity-induced levels. Only RIα increased after the TrkB blockade during presynaptic stimulation indicating that the TrkB signalling is downregulating it during the stimulation condition and suggesting that it could be involved in the upregulation of the previously described presynaptic activity-induced phosphorylation function of the Cβ subunit over SNAP-25 and Synapsin-1 [3]. Exogenous h-BDNF decreased almost all the molecules except AKAP150 which was increased.

### Presynaptic activity regulation of mAChRs and its dependence on BDNF/TrkB pathway to regulate Synapsin-1 and SNAP-25 PKA-dependent phosphorylation

**Fig. 6:**
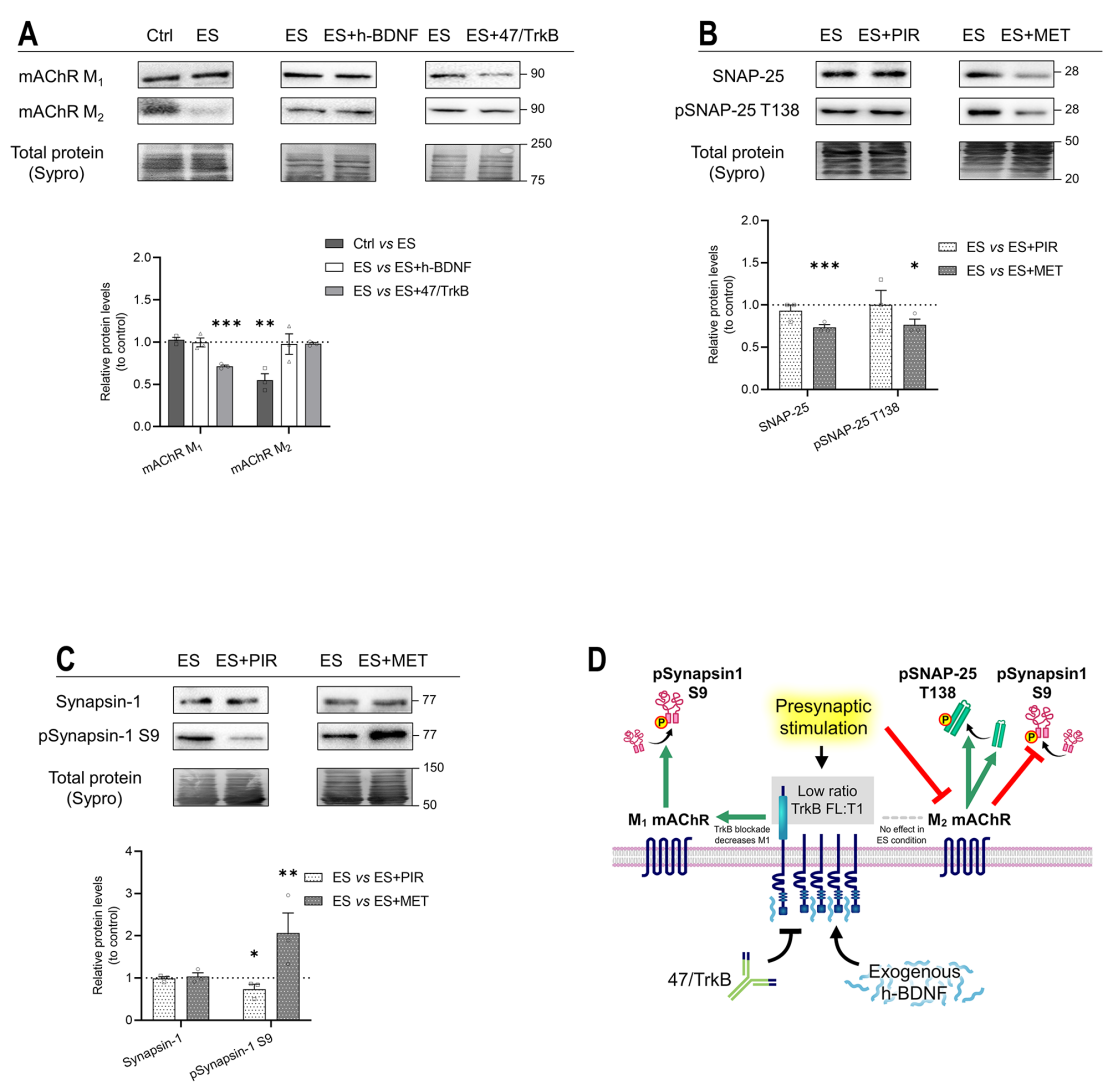
Modulation by exogenous h-BDNF or 47/TrkB of M_1_ and M_2_ mAChRs and the effects of M_1_ or M_2_ inhibition on targets of PKA SNAP-25 and Synapsin-1 protein levels during presynaptic activity. **A** Western blot analysis of M_1_ and M_2_ mAChRs protein levels after treatment with presynaptic stimulation without contraction —Ctrl versus ES—, with h-BDNF —ES versus ES + h-BDNF— and with 47/TrkB —ES versus ES + 47/TrkB. **B**,** C** Western blot analysis and data quantification of PKA targets protein levels in the diaphragm muscle after presynaptic activity with M_1_ or M_2_ mAChR inhibition by PIR —ES versus ES + PIR— or MET —ES versus ES + MET— respectively. **B** SNAP-25 and its phosphorylated form pSNAP-25 T138. **C** Synapsin-1 and its phosphorylated form pSynapsin-1 S9. **D** Graphical representation of the results, effect of TrkB activity during presynaptic stimulation on M_1_ and M_2_ mAChR and its following regulation of PKA targets. Data are expressed relative to experimental control – ES (dotted line) (mean ± SEM). *p < 0.05, **p < 0.01, and ***p < 0.001 versus the corresponding control. ES, electrical stimulation; mAChR, muscarinic Acetylcholine receptor; h-BDNF, exogenous recombinant human Brain-Derived Neurotrophic Factor; 47/trkB, anti-TrkB antibody clone 47/TrkB; PIR, Pirenzepine dihydrochloride (M_1_ mAChR selective agonist); MET, Methoctramine tetrahydrochloride (M_2_ mAChR selective agonist)

Due to the complexity of retrograde regulation and involvement of several pathways, for a better understanding of the effect of PKA activity on SNAP-25 and Synapsin-1 under synaptic activity conditions, we have investigated the involvement of activity-dependent muscarinic retrograde signalling because of the close relationship between PKA and mAChRs at the NMJ [1, 7] and the relationship between them and the activity-induced BDNF [24]. Firstly, we studied the effect of nerve-induced activity on the presynaptic mAChRs, M_1_ and M_2_, and, secondly, the presynaptic activity-dependence of the muscarinic receptors on the TrkB signalling (Fig. 6A). The results demonstrated that M_1_ levels were not modified as a response to the increase in presynaptic activity (Ctrl vs ES) while M_2_ was decreased. When TrkB pathway was activated with exogenous h-BDNF (ES vs ES + h-BDNF) or inhibited by 47/TrkB (ES vs ES + 47/TrkB), we found that only M_1_ was significantly downregulated by the blockade of the endogenous activity of TrkB during presynaptic activity.

In addition, we detected that the targets of PKA, SNAP-25 (Fig. 6B) and Synapsin-1 (Fig. 6C) were differently regulated by the presynaptic mAChRs in the presence of presynaptic activity. M_1_ (ES vs ES + PIR) upregulated only pSynapsin-1 S9 phosphorylation while M_2_ (ES vs ES + MET) promoted both total SNAP-25 levels and its phosphorylated form but downregulated pSynapsin-1 S9 phosphorylation. Fig. 6D shows a graphical summary of the findings.

In summary, the results show the involvement of the activity-dependent muscarinic receptors in the regulation of the phosphorylation of presynaptic PKA targets, Synapsin-1 and SNAP-25, and contribute to a better understanding of the TrkB regulation of these targets.

### Muscle contraction-induced BDNF/TrkB pathway affects PKA and its targets

**Fig. 7:**
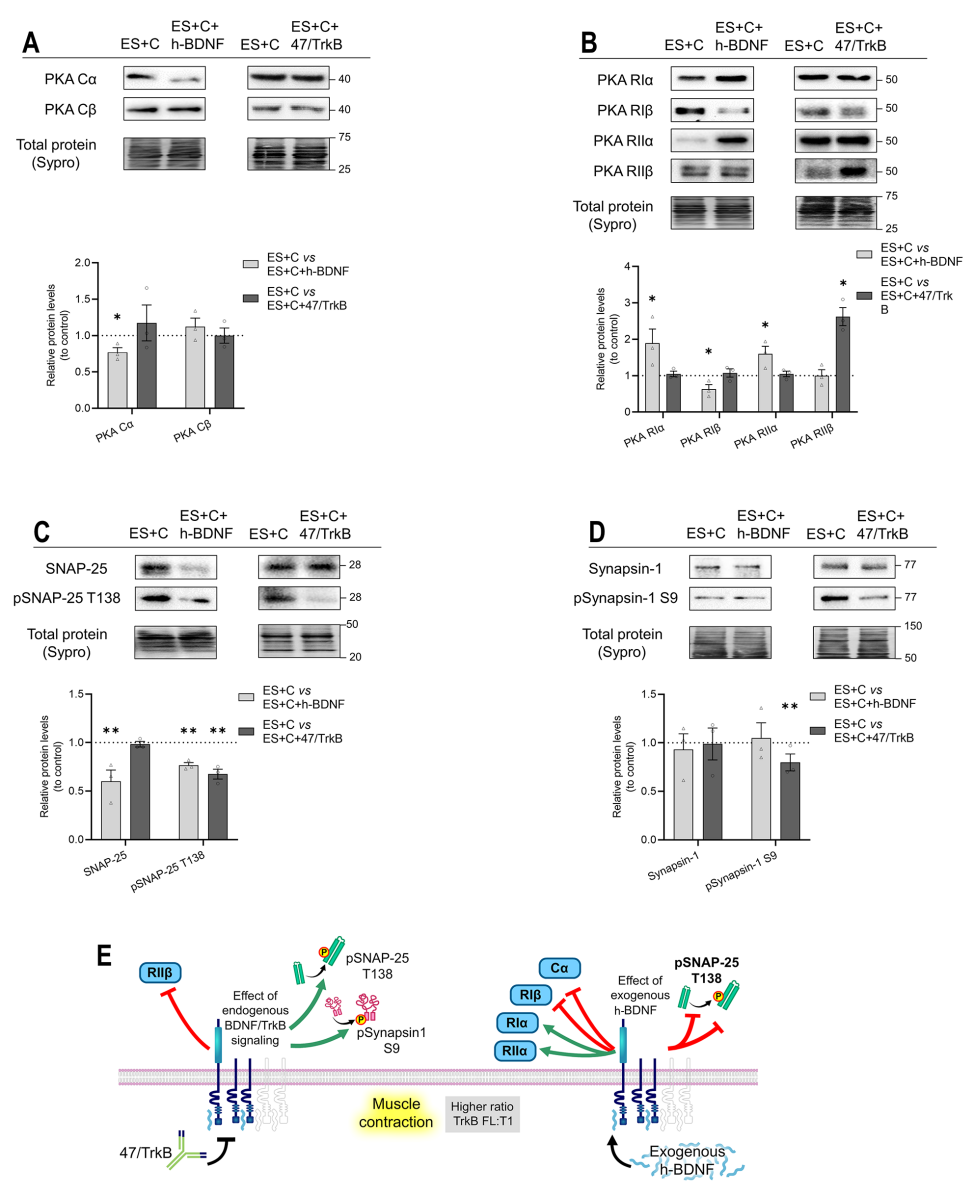
Modulation by exogenous h-BDNF or 47/TrkB of PKA catalytic and regulatory subunits and its targets SNAP-25 and Synapsin-1 protein levels during nerve-induced muscle contraction. **A-D** Western blot analysis of protein levels after treatment with nerve-induced muscle contraction with h-BDNF —ES + C versus ES + C + h-BDNF— and with 47/TrkB —ES + C versus ES + C + 47/TrkB. **A** Catalytic PKA Cα and Cβ subunits. **B** Protein kinase A regulatory subunit Iα/Iβ/IIα/IIβ. **C** SNAP-25 and its phosphorylated form pSNAP-25 T138. **D** Synapsin-1 and its phosphorylated form pSynapsin-1 S9. Data are expressed relative to experimental control – ES + C (dotted line) (mean ± SEM). *p < 0.05, **p < 0.01, and ***p < 0.001 versus the corresponding control. **E** Graphical representation of the results, effect of endogenous TrkB activity and exogenous h-BDNF during presynaptic stimulation with muscle contraction on PKA subunits and its targets. ES + C, electrical stimulation with muscle contraction; Cα/β, Protein kinase A Catalytic subunit α/β; RIα/RIβ/RIIα/RIIβ, Protein kinase A Regulatory subunits Iα/Iβ/IIα/Iiβ; h-BDNF, exogenous recombinant human Brain-Derived Neurotrophic Factor; 47/trkB, anti-TrkB antibody clone 47/TrkB

We next studied how the inhibition and activation of the TrkB receptor affect the PKA pathway under nerve-induced muscle contraction conditions. The TrkB receptor inhibition under muscle contraction ((ES + C) vs (ES + C) + 47/TrkB) did not change the levels of any PKA catalytic subunit (Fig. 7A). On the contrary, exogenous h-BDNF under muscle contraction ((ES + C) vs (ES + C) + h-BDNF) decreased Cα subunit.

Blocking the TrkB pathway under muscle contraction increases the PKA RIIβ without affecting RIα, RIβ and RIIα (Fig. 7B). Interestingly, exogenous h-BDNF under muscle contraction affected the regulatory subunits which were not altered during TrkB inhibition: RIα and RIIα were unregulated but RIβ was downregulated. At the same time, h-BDNF did not result in any change in RIIβ. This indicates that BDNF/TrkB receptor pathway can affect all regulatory subunits during contraction.

TrkB receptor blockade under muscle contraction treatment decreased pSNAP-25 T138 and pSynapsin-1 S9 protein levels without affecting their total proteins (Fig. 7C, D). Therefore, TrkB receptor pathway is necessary to induce pSNAP-25 T138 and pSynapsin-1 S9 phosphorylation during the normal function of the NMJ. When exogenous h-BDNF is added to the diaphragm during muscle contraction, SNAP-25 and its phosphorylated form are further diminished. However, Synapsin-1 was not affected by exogenous h-BDNF. This indicates that the action of the BDNF is to restrict the levels and PKA-phosphorylation of SNAP-25. Fig. 7E shows the graphical representation of the results.

**Fig. 8:**
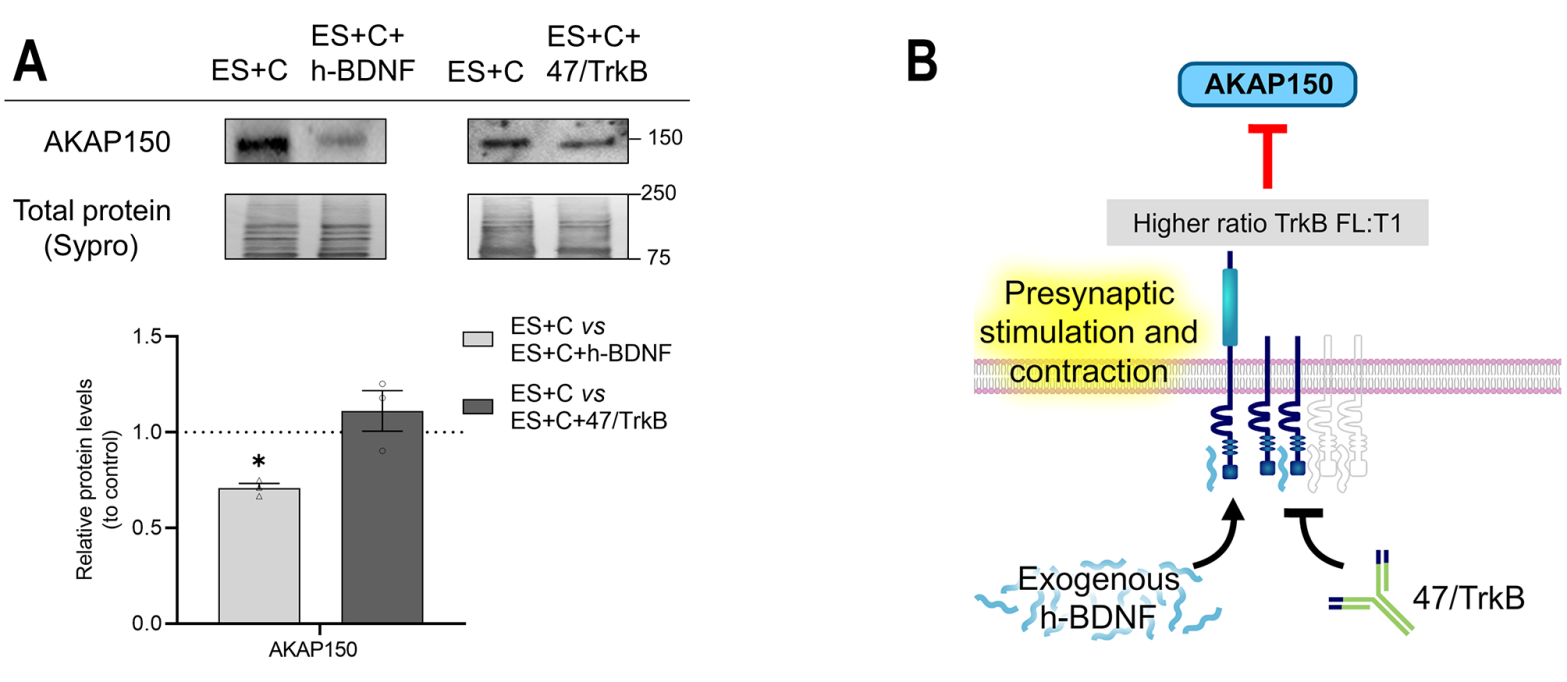
Modulation by exogenous h-BDNF or 47/TrkB of AKAP150 protein levels during nerve-induced muscle contraction. **A** Western blot analysis and data quantification of AKAP150 protein levels in the diaphragm muscle after nerve-induced muscle contraction with h-BDNF or 47/TrkB incubation. Data are expressed relative to experimental control – ES + C (dotted line) (mean ± SEM). *p < 0.05, **p < 0.01, and ***p < 0.001 versus the corresponding control. **B** Graphical representation of the results, effect of endogenous TrkB activity and exogenous h-BDNF during presynaptic stimulation with muscle contraction on AKAP150. AKAP150, A-kinase anchor protein 150; ES + C, electrical stimulation with muscle contraction; h-BDNF, exogenous recombinant human Brain-Derived Neurotrophic Factor; 47/trkB, anti-TrkB antibody clone 47/TrkB

The addition of 47/TrkB during muscle contraction did not change AKAP150 protein levels (Fig. 8A). Exogenous h-BDNF in the same condition decreased the AKAP150 protein levels, indicating that AKAP150 can be further modulated in enhanced BDNF conditions. The effects are represented in the graphical model (Fig. 8B).

In summary, the TrkB receptor effect in muscle contraction conditions shows an important increase of SNAP-25 and Synapsin-1 PKA-induced phosphorylation in parallel with the decrease in RIIβ levels, indicating the involvement of BDNF/TrkB pathway in the regulation of catalytic activity of PKA through RIIβ regulatory subunits. The PKA Cα catalytic subunit can be considered constitutive and independent of both the neuromuscular activity and BDNF/TrkB pathway, as neither synaptic activity nor TrkB pathway modulations affect its levels. In contrast, the Cβ subunit shows a highly controlled regulation that influences the phosphorylation of the PKA targets analysed.

### Muscle contraction regulation of mAChRs and its dependence on BDNF/TrkB pathway to regulate Synapsin-1 and SNAP-25 PKA-dependent phosphorylation

**Fig. 9:**
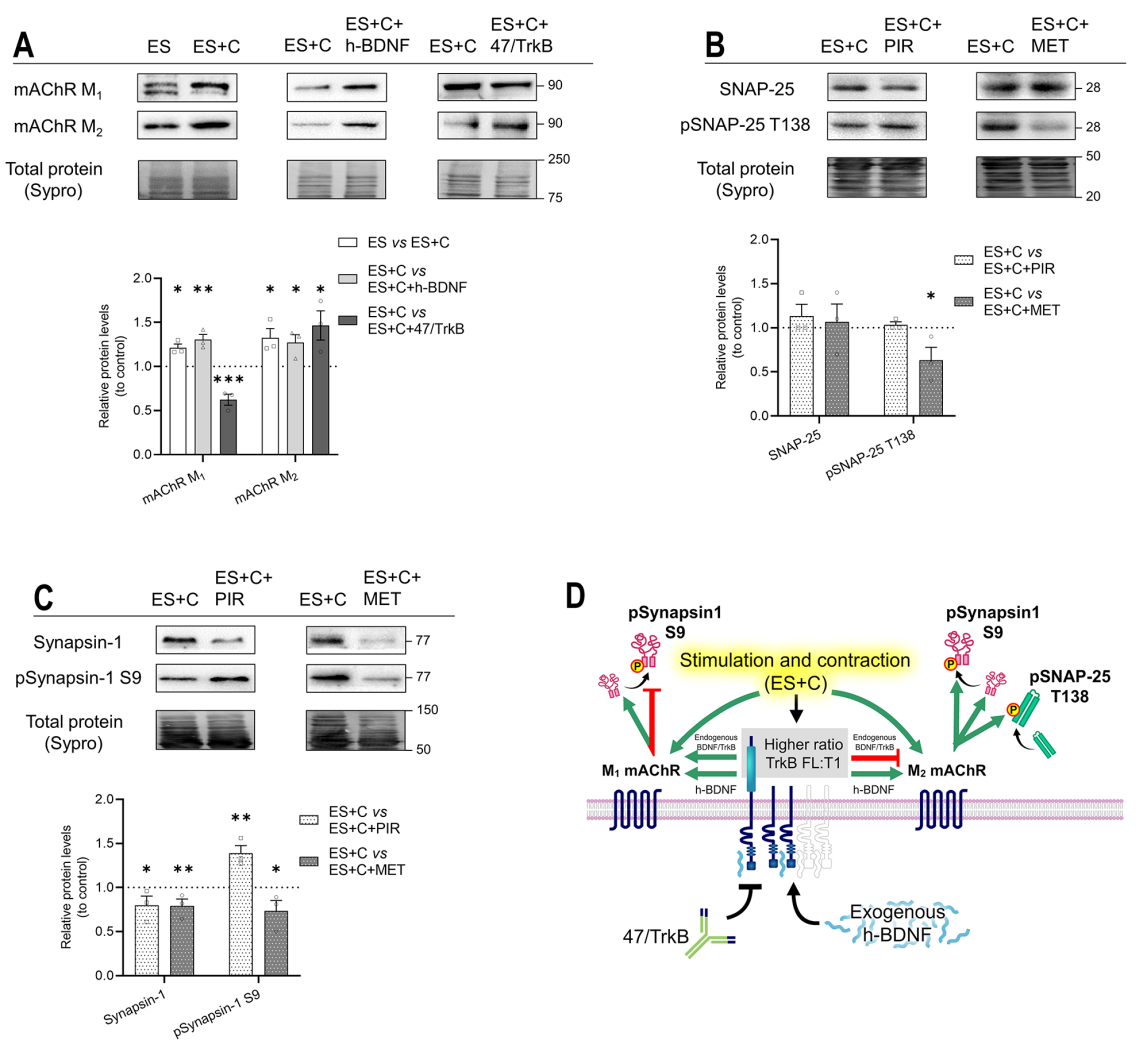
Modulation by exogenous h-BDNF or 47/TrkB of M_1_ and M_2_ mAChRs and the effects of M_1_ or M_2_ inhibition on targets of PKA SNAP-25 and Synapsin-1 protein levels during nerve-induced muscle contraction. **A** Western blot analysis of M_1_ and M_2_ mAChRs protein levels after treatment with presynaptic stimulation with muscle contraction —ES versus ES + C—, with h-BDNF —ES + C versus ES + C + h-BDNF— and with 47/TrkB —ES + C versus ES + C + 47/TrkB. **B**,** C** Western blot analysis and data quantification of PKA targets protein levels in the diaphragm muscle after presynaptic activity with M_1_ or M_2_ mAChR inhibition by PIR —ES + C versus ES + C + PIR— or MET —ES + C versus ES + C + MET— respectively. **B** SNAP-25 and its phosphorylated form pSNAP-25 T138. **C** Synapsin-1 and its phosphorylated form pSynapsin-1 S9. **D** Graphical representation of the results, effect of TrkB activity during presynaptic stimulation with muscle contraction on M_1_ and M_2_ mAChR and its following regulation of PKA targets. Data are expressed relative to experimental control – ES + C (dotted line) (mean ± SEM). *p < 0.05, **p < 0.01, and ***p < 0.001 versus the corresponding control. ES + C, electrical stimulation with muscle contraction; mAChR, muscarinic Acetylcholine receptor; h-BDNF, exogenous recombinant human Brain-Derived Neurotrophic Factor; 47/trkB, anti-TrkB antibody clone 47/TrkB; PIR, Pirenzepine dihydrochloride (M_1_ mAChR selective agonist); MET, Methoctramine tetrahydrochloride (M_2_ mAChR selective agonist)

Figure 9A shows the nerve-induced muscle contraction regulation of the presynaptic mAChRs, M_1_ and M_2_, and the dependence of these muscarinic receptors on the TrkB signalling in the muscle contraction conditions. Nerve-induced muscle contraction (ES vs ES + C) and exogenous h-BDNF ((ES + C) vs (ES + C) + h-BDNF) upregulated both M_1_ and M_2_ mAChRs. TrkB was differently regulating mAChRs ((ES + C) vs (ES + C) + 47/TrkB), upregulating M_1_ and downregulating M_2_ respectively.

Figures 9B and C show the regulation of M_1_ and M_2_ on the PKA targets, SNAP-25 (Fig. 9B) and Synapsin-1 (Fig. 9C), in the nerve-induced contraction condition. M_2_ ((ES + C) vs (ES + C) + MET) upregulated phosphorylation of SNAP-25 T138 and both Synapsin-1 and pSynapsin-1 S9. At the same time, M_1_ ((ES + C) vs (ES + C) + PIR) upregulated Synapsin-1 but downregulated its phosphorylated form.

All the findings are summarized on the graphical representation figure (Fig. 9D).

In summary, our observations indicate that, in the muscle contraction condition, M_2_ facilitates the phosphorylation of Synapsin-1 at the S9 site, while M_1_ diminishes it. Furthermore, TrkB signalling, activated during muscle contraction, upregulates M_1_ and concurrently downregulates M_2_. Consequently, the primary impact of muscle contraction appears to be mediated via M_1_ mAChR, leading to a reduction in phosphorylated Synapsin-1 at the S9 site.

## Discussion

**Fig. 10:**
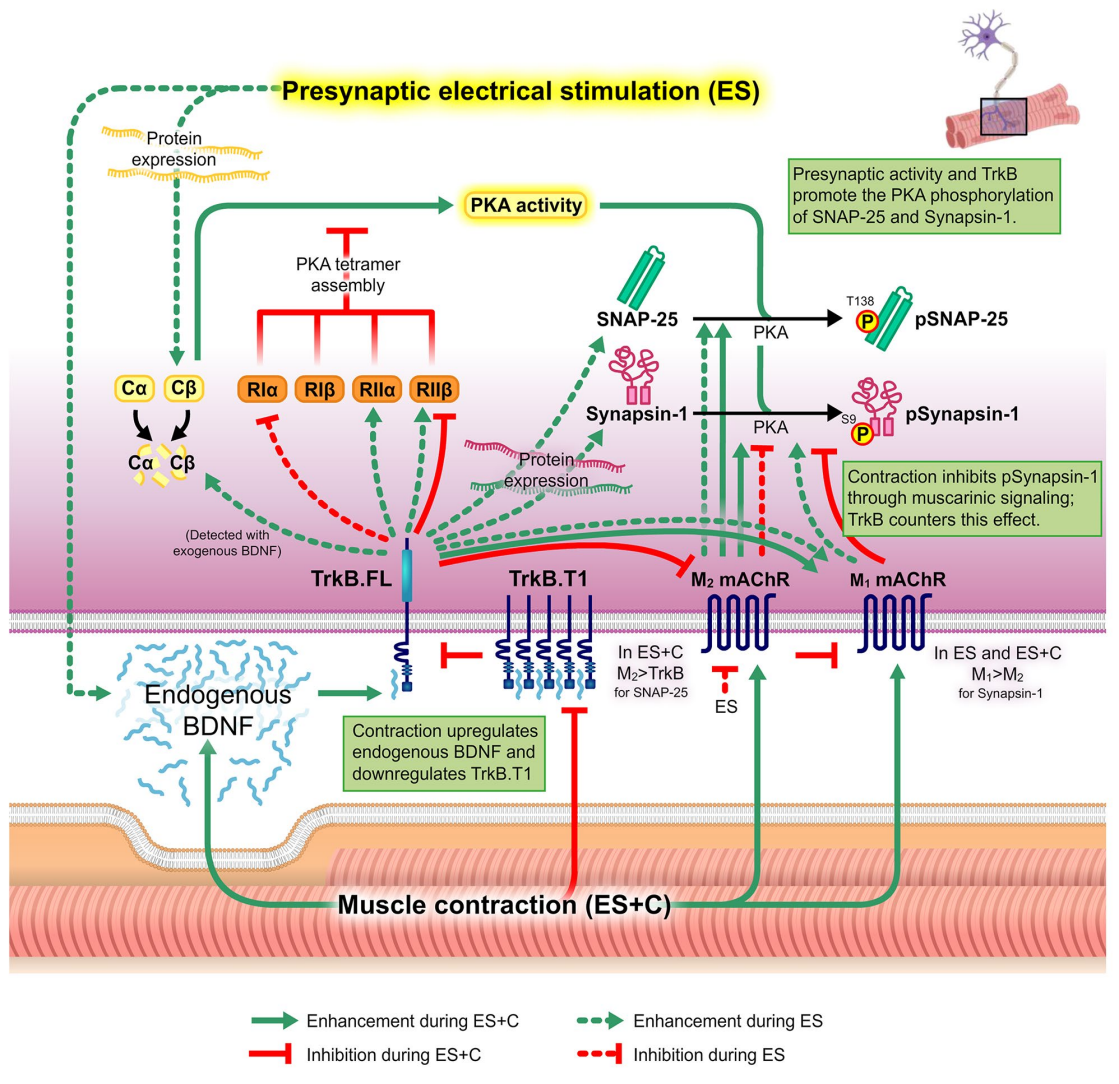
Summary of PKA subunits and its targets regulation by BDNF/TrkB and muscarinic signalling at the NMJ during presynaptic activity and nerve-induced muscle contraction. Model of the PKA regulation resulted from this study during presynaptic activity and nerve-induced muscle contraction

BDNF promotes the survival of neurons in the central nervous system and is enhanced by synaptic activity [49]. In the peripheral nervous system, BDNF is a contraction-inducible molecule [10, 11] that, by binding to TrkB in the nerve terminal membrane, contributes to the retrograde neuroprotective control done by muscles, which is necessary for the proper function of motor neurons [12, 50]. BDNF/TrkB signalling retrogradely triggers downstream presynaptic pathways, essential for synaptic function and maintenance [8, 12, 24, 51, 52], but until now it was unknown how it regulates the PKA subunits and its targets during NMJ activity. Previously we demonstrated the retrograde muscle contraction effects over PKA subunits and AKAP150 and their interactions to regulate ACh release at the NMJ through the phosphorylation of pSNAP-25 T138 and pSynapsin-1 S9 [3]. In the present work, we reveal that the activity-dependent BDNF/TrkB signalling regulates muscarinic receptors and PKA to balance synaptic function, determining that this regulation is different if induced by presynaptic stimulus or by postsynaptic muscle contraction. Figure 10 summarizes the results.

### Presynaptic activity-induced TrkB-PKA regulation and the involvement of muscarinic signalling

In the following sections, we distinguish two BDNF modulations: the effect of endogenous BDNF, observed when blocking TrkB with 47/TrkB, and the effect of adding exogenous h-BDNF. Both treatments have been studied during presynaptic electrical stimulation (ES) conditions and during electrical stimulation resulting in contraction (ES + C). The PKA Cβ subunit level is activity-dependent and increases after nerve electrical stimulation [3]. Blocking TrkB during ES does not affect Cβ levels, indicating that Cβ is not dependent on the endogenous BDNF/TrkB activity, possibly due to the predominance of TrkB.T1 isoform in this condition. Other signalling pathways could influence the levels of this subunit, including its regulation by the PKA regulatory subunits, which is analysed below. However, adding exogenous h-BDNF during ES decreases the Cβ subunit, showing some responsiveness to BDNF/TrkB, probably due to activity-dependent degradation. On the other hand, a contraction-induced decrease in the Cβ subunit has been demonstrated in parallel with enhanced levels of endogenous BDNF and an increase in the TrkB.FL/TrkB.T1 ratio [3, 6]. This would be consistent with the inhibitory effect of exogenous h-BDNF on Cβ during ES that could be mediated by TrkB.FL.

The protein levels of PKA regulatory (R) subunits influence the activity of the Cβ subunit, with an inverse relationship [7, 53–55]. A good candidate for PKA regulation by TrkB could be RIα, as we found that TrkB decreases RIα protein levels, and this would allow for increased catalytic activity of PKA Cβ and the observed upregulation of pSNAP-25 T138. At the same time, we have shown that AKAP150 activity is promoted by TrkB receptor and enhanced by exogenous h-BDNF. Therefore, AKAP150 might regulate the PKA catalytic activity by binding regulatory subunits. Taking into consideration our previous results, RIα could be responsible, as it shows concordance with AKAP150 regulation [3], and its levels are regulated by the BDNF/TrkB pathway. On the other hand, TrkB signalling in ES increases RIβ and RIIα, which would reduce PKA activity. Although the two substrates studied here correlate with enhanced PKA activity, we cannot discard that these R subunits might affect other PKA substrates.

We have analysed the activity of the Cβ subunit by studying the phosphorylation of two PKA targets from the nerve terminal of the NMJ: SNAP-25 and Synapsin-1. Presynaptic activity does not change PKA-dependent pSNAP-25 T138 [3] but enhances PKC-dependent pSNAP-25 S187 [9]. Thus, there might be a balance between the different mechanisms that could control these phosphorylations and, therefore, the SNAP-25 function in vesicle exocytosis. We previously determined that pSNAP-25 S187 is independent of TrkB activity [9]. Here we show that pSNAP-25 T138 is TrkB-dependent, indicating a specific regulation of the molecule by TrkB.

To further understand the relation between PKA phosphorylation and TrkB, we have studied the interaction of the muscarinic receptor in these molecular pathways as the mAChRs regulate PKA in basal conditions [7] and TrkB and mAChRs interact in the ACh release at the NMJ [24]. We analysed the activity-dependence of the muscarinic receptors and their regulation by the TrkB receptor. Our results demonstrate an activity-dependence regulation of the two presynaptic muscarinic receptors that are active in the adult NMJ, M_1_ and M_2_. M_2_ is downregulated by presynaptic activity while both M_1_ and M_2_ are upregulated by nerve-induced muscle contraction. In the present work, we observed no change in M_1_ mAChR under presynaptic stimulation. However, inhibiting TrkB in this condition resulted in reduced M_1_ mAChR levels, suggesting that TrkB sustains M_1_ mAChR and likely enhances ACh release through this receptor, aligning with prior functional observations [5, 24]. Furthermore, blocking the TrkB receptor during ES, when the T1 isoform is predominant [10], is functionally associated with a reduction of ACh release [24] and here we found a decrease in M_1_ mAChR, which associated with enhancement of ACh release [24], without impact on M_2_ mAChR. Notably, TrkB needs to be active for presynaptic stimulation to reduce M_2_ mAChR [24]. This interplay between receptors provides insight into how inhibiting TrkB could diminish ACh release. Furthermore, these findings align with prior research demonstrating that TrkB enhances PKC signalling at the NMJ under presynaptic stimulus conditions [8–10] to promote ACh release.

In addition, we analysed the dependence of PKA phosphorylation on M_1_ and M_2_ mAChRs during presynaptic activity. Under synaptic activity conditions, pSNAP-25 T138 is dependent on M_2_ regulation. Previous results show that, in basal conditions, M_1_ regulates PKC-dependent pSNAP-25 S187 [22] while M_2_ regulates PKA-dependent pSNAP-25 T138 [7]. The present study also identified a link between the M_2_ regulation of PKA-dependent pSNAP-25 T138 phosphorylation in the presynaptic activity condition, thereby confirming the previously established association. Altogether, indicates a complex balance between the regulation of muscarinic receptors to control SNAP-25 function through PKC and PKA.

Regarding the dependence of the muscarinic receptors from BDNF/TrkB signalling, we observe that TrkB enhances M_1_ during presynaptic activity and this is coincident with an M_1_ upregulation of pSynapsin-1 S9. This signalling could be responsible for the increase of pSynapsin-1 S9 during presynaptic activity explained in our previous research [3]. When we analysed the regulation of Synapsin-1 by TrkB in the same condition of activity, we found that TrkB upregulates both total levels of Synapsin-1 and pSynapsin-1 S9. Other pathways could influence this regulation. For example, our results show that M_1_ mAChR promotes phosphorylation of Synapsin-1 S9 and M_2_ mAChR downregulates it. To interpret these two complementary activities, we should consider that M_2_ blockade increases M_1_ protein levels [7]. Thus, the effect of MET might include an enhanced activity of M_1_ receptors, and the results obtained from the experiments with PIR confirm it. Because ES reduces M_2_ mAChR protein levels we can see the increase of pSynapsin-1 S9. Overall, muscarinic signalling is increasing the level of pSynapsin-1 S9. This is coincident with electrophysiology results earlier demonstrated in our laboratory showing that PKA and balance of M_1_/M_2_ enhance ACh release [26, 56], as pSynapsin-1 S9 does [57, 58]. M_2_ mAChR decreases the ACh release and the current results indicate that it decreases pSynapsin-1 S9. When we apply the presynaptic activity, we can see the effect of the downregulation of M_2_ mAChR and the increase of ACh release would be maintained by phosphorylation of pSynapsin-1 S9. Phosphorylation of pSynapsin-1 S9 is upregulated by M_1_ and, in turn, TrkB increases M_1_ to increase ACh release. Together, the results indicate a well-balanced relationship between M_1_/M_2_ and its regulation by TrkB to regulate PKA targets involved in synaptic vesicle exocytosis.

By analysing the effect of exogenous h-BDNF we have found the decrease of both Synapsin-1 and pSynapsin-1 S9. The modulation of this synaptic protein, and SNAP-25, may be influenced by mTOR activity. As the promotion of mTOR signalling by BDNF has been demonstrated [59, 60], this signalling pathway could be facilitated by endogenous BDNF acting through TrkB at the NMJ. Therefore, the decrease of both SNAP-25 and Synapsin-1 and their phosphorylated forms with exogenous h-BDNF could be linked to a downregulation of the protein synthesis during the interaction of BDNF/TrkB pathway and mTOR signalling. As discussed in the next section, muscle contraction has a similar effect to adding exogenous h-BDNF during presynaptic stimulus. This is because muscle contraction releases BDNF and decreases Synapsin-1 and pSynapsin-1 S9 levels [3].

### Muscle contraction induced-TrkB-PKA regulation and the involvement of muscarinic signalling

Nerve-induced muscle contraction strongly regulates PKA subunits’ dynamics to enhance pSNAP-25 T138 [3] and, at the same time, enhances BDNF expression and downregulates TrkB.T1 [10]. However, we did not know until now that both mechanisms are related. In brief, muscle contraction promotes Cβ activity. Additionally, contraction upregulates AKAP150, which recruits enough RIIβ regulatory subunits to permit Cβ activity and enhance pSNAP-25 T138 phosphorylation [3]. Here we show that Cβ levels are not directly dependent on endogenous TrkB activity in the contraction condition because the levels of Cβ did not change with 47/TrkB. However, TrkB downregulates the RIIβ regulatory subunit, resulting in an increase in the Cβ activity that is manifested in the PKA target, pSNAP-25 T138. Together, our results show that the TrkB receptor isoforms’ ratio regulates RIIβ: when it is favourable to T1 (by presynaptic activity) upregulates RIIβ, whereas when it is favourable to FL (by contraction activity) downregulates RIIβ. Furthermore, this Cβ and RIIβ regulation by TrkB is in concordance with the TrkB regulation over PKA-induced SNAP-25 phosphorylation on T138 site. Thus, TrkB is one of the regulators of SNAP-25 phosphorylation through PKA activity although TrkB does not regulate the PKC-dependent SNAP-25 phosphorylation on S187 site [9]. This mechanism implies a minimum remainder ACh release during muscle contraction, ensuring the maintenance of contraction if required. This is in concordance with the roles of pSNAP-25 T138 in controlling the size of releasable vesicle pools and pSNAP-25 S187 in refilling the emptied vesicle pools [30] through neurotransmitter release enhanced by its nPKCε phosphorylation [9]. In addition, this TrkB regulation on RIIβ to enhance pSNAP-25 T138 acts in addition to the M_2_ muscarinic receptor regulation on the pSNAP-25 T138, as nerve-induced muscle contraction enhances M_2_ muscarinic receptor, which upregulates pSNAP-25 T138. That adds new activity-dependent regulation knowledge to the previously explored association between M_2_ mAChR and PKA-dependent SNAP-25 phosphorylation in the basal condition [7]. Although TrkB downregulates M_2_ during muscle contraction condition, the contraction-induced enhancement of M_2_ seems stronger.

Exogenous h-BDNF caused a decrease in pSNAP-25 T138 levels, similar to the one detected in the presynaptic condition. It is surprising that, once again, the effect of exogenous h-BDNF is similar to the effect of the inhibitor 47/TrkB. Adding exogenous h-BDNF could act through pathways other than TrkB.FL receptors and promote PKA activity over pSNAP-25 T138. For example, BDNF could affect SNAP-25 through mTOR. It is known that the mTORC1 pathway promotes protein synthesis while inhibiting proteolysis [61–63]. Also, there are several evidence that relate mTOR with BDNF [59, 60]. According to this, we can hypothesize that exogenous h-BDNF could affect the synthesis of SNAP-25 through mTORC1 pathway. This interesting regulation requires further investigation.

Nerve-induced muscle contraction has the opposite effect on Synapsin-1 than on SNAP-25 PKA-phosphorylation, as it downregulates levels of Synapsin-1 and pSynapsin-1 S9 at the NMJ [3]. In the present work, we demonstrate that TrkB opposes this action and promotes the phosphorylation of Synapsin-1 S9 during contraction. Cβ subunit of PKA could be responsible for this, as TrkB regulates Cβ activity through decreasing RIIβ. Therefore, the action of TrkB would counter and balance Synapsin-1 phosphorylation levels during contraction. The nerve-induced muscle contraction might downregulate pSynapsin-1 S9 through other pathways. For example, CaMKI/IV pathway is known to phosphorylate pSynapsin-1 on S9 [32, 33]. On the other hand, the muscarinic mAChR pathway could be also involved, as our results show that both M_1_ and M_2_ subtypes of mAChRs are enhanced during muscle contraction. In addition, we have found that M_2_ promotes the phosphorylation of Synapsin-1 S9 and M_1_ decreases the level of pSynapsin-1 S9. TrkB signalling during muscle contraction upregulates M_1_ and downregulates M_2_. Thus, the main effect of muscle contraction would be conveyed through M_1_ mAChR, which would decrease pSynapsin-1 S9.

The exogenous h-BDNF does not affect the level of pSynapsin-1 S9, indicating that during nerve-induced muscle contraction, it has reached the maximal level of BDNF that could affect the pSynapsin-1 S9.

### Presynaptic location of the mechanism

TrkB receptor is present in the three cells forming the NMJ [64], suggesting that TrkB signalling could be initiated in any of these cells. In the same way, several subunits of PKA, including the Cβ subunit, have been located at the NMJ [7, 65, 66]. Thus, PKA signalling could occur in the three cells. Here, however, we evaluated presynaptic PKA substrates, SNAP-25 and Synapsin-1, to show the TrkB-PKA regulation at the nerve terminal of the NMJ, indicating that the proposed signalling is presynaptic. However, we cannot discard that the activity-dependent effect of TrkB on PKA may also affect postsynaptic PKA targets and be part of the retrograde signalling pathway described here. Indeed, PKA has important postsynaptic roles, such as regulating the stability of nicotinic AChRs at the NMJ together with PKC [39, 67, 68], a mechanism sensitive to synaptic activity [69].

## Conclusion

BDNF/TrkB signalling is a crucial element of the activity-dependent bidirectional communication between nerve terminals and myocytes for its responsibility in the retrograde regulation of neurotransmission at the NMJ. In this synapse, mAChRs autocrinally modulate ACh release and PKA activity amplifies it. In the current study, our findings highlight the activity-dependent role of TrkB to regulate the muscarinic receptors, and the dynamics of PKA subunits, enhancing the phosphorylation of key targets involved in synaptic vesicle exocytosis, such as SNAP-25 and Synapsin-1. Figure 10 shows a summary of the TrkB/mAChRs/PKA regulation over pSNAP-25 T138 and pSynapsin-1 S9 that occurs during presynaptic activity and nerve-induced muscle contraction.

**Presynaptic-induced** action of TrkB upregulates the level of SNAP-25 and, facilitates its phosphorylation at T138 by reducing RIα levels, enhancing PKA catalytic activity and, subsequently, promoting pSNAP-25 T138 level, which is dependent exclusively on M_2_. This TrkB upregulation also extends to total and phosphorylated levels of pSynapsin-1 S9, possibly influenced by RIα and the muscarinic pathway, with a predominant M_1_ action over M_2_, as M_1_ mAChR was promoted by the activity of BDNF/TrkB pathway and M_2_, that opposes to the phosphorylation, was downregulated by the presynaptic stimulus. However, during **nerve-induced muscle contraction**, TrkB downregulates PKA RIIβ activity and upregulates AKAP150 levels, which may facilitate Cβ subunit catalytic activity and promote phosphorylation of SNAP-25 and Synapsin-1. Muscarinic receptors, particularly M_1_, have the main influence over the phosphorylation of Synapsin-1 decreasing the level of pSynapsin-1 S9, as activity of M_1_ mAChR was enhanced and activity of M_2_ was diminished by the BDNF/TrkB signalling. However, phosphorylation of SNAP-25 exclusively depends on M2.

Together, these results contribute to a better understanding of the coordinated activity-dependent bidirectional communication between nerve terminals and muscle fibres to maintain an optimal acetylcholine release through PKA-phosphorylation targets at the NMJ. These new findings have the originality and great relevance of interrelating two fundamental pathways in synaptic modulation: one retrograde (neurotrophic) and the other autocrine (muscarinic). This cooperation between signalling pathways highlights the complexity of synaptic regulation that includes several other mechanisms such as purinergic, peptides (e.g. CGRP), etc. It is important to obtain clear molecular information on each of them to be able to integrate and relate each particular function in the integral regulation mechanism that gives plasticity to synapses.

This complex interplay assumes particular importance in the context of neuromuscular diseases marked by impaired crosstalk between nerves and muscles. The identified molecular pathways not only deepen our fundamental understanding of neuromuscular physiology but also hold the potential for targeted therapeutic interventions to address conditions characterized by disrupted neuromuscular communication. That can be the case of NMJs affected by amyotrophic lateral sclerosis, in which TrkB is impaired [23] and pharmacological activation of muscarinic receptors could transactivate TrkB to assure neurotransmission.

### Electronic supplementary material

Below is the link to the electronic supplementary material.

Supplementary Material 1

## Data Availability

No datasets were generated or analysed during the current study.

## Abbreviations

Ach: Acetylcholine
AKAP150: A-kinase anchoring protein 150
BDNF: Brain-derived neurotrophic factor
Cβ: Catalytic beta subunit of Protein kinase A
CaMKI/IV: Calmodulin-dependent protein kinase I/IV
EPP: Evoked endplate potentials
nAChR: Nicotinic Acetylcholine receptor
mAChR: Muscarinic Acetylcholine receptor
M_1_: M_1_-type muscarinic Acetylcholine receptor
M_2_: M_2_-type muscarinic Acetylcholine receptor
MET: Methoctramine
mTOR: Mammalian target of Rapamycin
NMJ: Neuromuscular junction
NT-4: Neurotrophin 4
PIR: Pirenzepine
PKA: Protein kinase A
PKC: Protein kinase C
RIα/RIβ/RIIα/RIIβ: Protein kinase A Regulatory subunit Iα, Iβ, IIα or IIβ
SNAP-25: Synaptosomal-associated protein of 25 kDa
TrkB: Tropomyosin-related kinase B receptor
VDSC: Voltage-dependent sodium channels
